# Formyl Peptide Receptors and Annexin A1: Complementary Mechanisms to Infliximab in Murine Experimental Colitis and Crohn’s Disease

**DOI:** 10.3389/fimmu.2021.714138

**Published:** 2021-09-17

**Authors:** Marina de Paula-Silva, Gustavo Henrique Oliveira da Rocha, Milena Fronza Broering, Maria Luíza Queiroz, Silvana Sandri, Rodrigo Azevedo Loiola, Sonia Maria Oliani, Andrea Vieira, Mauro Perretti, Sandra Helena Poliselli Farsky

**Affiliations:** ^1^Department of Clinical and Toxicological Analyses, University of São Paulo (USP), São Paulo, Brazil; ^2^Centre for Biochemical Pharmacology, The William Harvey Research Institute, Barts and The London School of Medicine, Queen Mary University of London (QMUL), London, United Kingdom; ^3^Gastroenterology Service, Irmandade da Santa Casa de Misericórdia de São Paulo, São Paulo, Brazil; ^4^Department of Biology, São Paulo State University (UNESP), São José do Rio Preto, São Paulo, Brazil

**Keywords:** biomarkers, formyl peptide receptor, annexin A1, infliximab, Crohn’s disease, dextran sodium sulfate

## Abstract

Non-responsiveness to anti-TNF-α therapies presents relevant rates in inflammatory bowel disease patients, presenting the need to find biomarkers involved in therapeutic efficacy. Herein, we demonstrate that higher levels of colonic formyl peptide receptor 1 and annexin A1 correlate with histological recovery in Crohn’s disease patients under remission. Using the dextran sulfate sodium colitis model in mice, we suggest that infliximab induces annexin A1 expression and secretion in activated intestinal leukocytes. Conversely, this mechanism might stimulate epithelial formyl peptide receptors, inducing wound healing and consequent histological remission. Our data indicate that assessing intestinal expressions of formyl peptide receptors and annexin A1 might provide precious information on the disease activity and responsiveness to infliximab in inflammatory bowel disease patients.

## Introduction

Inflammatory bowel diseases (IBDs), mainly Crohn’s disease (CD) and ulcerative colitis (UC), are characterized by severe gastrointestinal inflammation (1). Biological therapies, such as monoclonal antibodies and infliximab (IFX), are very effective in inducing remission for moderate-to-severe IBDs (2, 3). By binding soluble and transmembrane tumor necrosis factor alpha (TNF-α), IFX attenuates inflammation and decreases the need for surgery (2, 4, 5). However, side effects and non-responsiveness illuminate the relevance of validating biomarkers to assess therapeutic efficacy (6, 7).

We previously described a possible relationship between the response to IFX and the expression of annexin A1 (AnxA1) in mice with experimental colitis (8). AnxA1 is a resolutive mediator in human and experimental conditions such as cardiovascular diseases (9, 10), multiple sclerosis (11), and rheumatoid arthritis (12). AnxA1 is produced by epithelial cells and secreted by infiltrating leukocytes in IBD patients (13). Local expression of AnxA1 is pivotal to tissue recovery in CD and experimental colitis (14–16). Recently, it has been suggested that low levels of AnxA1 in CD support the uncontrolled inflammation that perpetuates the disease and that differential expressions of AnxA1 might allow the identification of disease severity patterns (14, 17). After cell activation, AnxA1 is mobilized to the membrane, where it is able to trigger anti-inflammatory pathways through formyl peptide receptors (FPRs) (18). FPRs have been increasingly studied in IBD as they participate in antimicrobial and inflammatory processes. FPR expression in the gut correlates with pathology during acute inflammation, but plays a protective role in the chronic phases (19). FPR1 is a wound closure mediator (20, 21), whereas FPR2 induces mucosal healing by regulating the traffic of leukocytes into the inflamed tissue (22, 23).

This background provides valuable information about the roles of AnxA1 in IBD; however, only a couple of studies—including ours—have addressed the involvement of AnxA1 in the efficacy of IFX (8, 14). Also, none has explored the potential participation of the AnxA1–FPR axis. Herein, we describe some IFX mechanisms that are affected by the AnxA1–FPR axis and seek to explain how it could mediate distinct responses to anti-TNF-α.

## Materials and Methods

### Patient Approach and Ethics Statement

CD patients from the Santa Casa School of Medical Sciences (São Paulo, Brazil) and donors with no IBD history willingly donated blood (12 ml). Medical records and paraffinized colon biopsies provided the CD Activity Index (CDAI) (24) and microscopic grading (25). This study was conducted in accordance with the Declaration of Helsinki and the ethics boards from Santa Casa and the University of São Paulo (protocol #07100819.3.0000.0067). Written consent was obtained.

### Criteria for Patient Enrollment

The diagnosis of CD for patients participating in this study took into account endoscopic, histological, and clinical criteria assessed and interpreted by members of the medical staff from the Santa Casa School of Medical Sciences. Individuals younger than 18 years and/or with a diagnosis of an infectious disease (such as tuberculosis, chlamydia, and the common flu) were excluded.

Participants were divided into groups as follows:

Blood donors: Healthy individuals with no prior history of IBD provided control samples of blood matching the average age, gender, and ethnicity from CD groups. “Remission patients” reached clinical remission upon IFX treatment, while “Failure patients” were refractory to previous therapies and were not responding clinically to IFX at the moment of blood donation. One untreated CD patient provided active disease parameters and was receiving other medications (including immunosuppressants and corticosteroids), but not IFX.

Biopsy donors: Remission patients reached clinical and histological remission when treated with IFX. The Failure group was composed of patients who did not present improvement of clinical parameters and histological homeostasis upon IFX. “CD untreated” individuals were those with an active disease despite treatment administration of other medications (including immunosuppressants and corticosteroids), but not IFX.

It should be noted that all patients enrolled in this study had previously received or were receiving other medication classes at the time of our sample collection. The absence/interruption of responsiveness to other therapies followed by remission or non-remission upon IFX was the defining parameter for separating the Remission and Failure groups, respectively. Clinical parameters (mean and range of age, gender, and concomitant medications) from the Untreated CD, CD+IFX Remission, or CD+IFX Failure groups are presented in **Table 1**.

**Table 1:** Characteristics from CD^a^ patients and healthy donors.

Parameters		Blood			Tissue		
	Healthy donors	CD patients					
		Untreated	Remission	Failure	Untreated	Remission	Failure
**Average age (mín.–max.)**	32 (23–41)	24	35 (19–50)	39 (19–63)	28 (19–49)	43 (17–44)	38 (61–16)
**Standard deviation (age)**	10.11	0.00	12.14	14.65	13.96	3.51	31.81
**Females**	3	1	8	5	3	2	0
**Males**	2	0	4	7	1	1	2
**Total**	5	1	12	11	4	3	2
**Medications**							
**Corticosteroids**	–	–	–	3 (27.3%)	1 (25%)	–	1 (50%)
**Mesalazine**	–	1 (100%)	3 (25%)	6 (54.6%)	2 (50%)	2 (66.6%)	1 (50%)
**Azathioprine**	–	–	3 (25%)	4 (36.4%)	3 (75%)	–	2 (100%)
**Anti-diarrheic**	–	–	1 (8.3%)	1 (9.1%)	–	1 (33.3%)	–
**Antidepressants**	2 (40%)	–	2 (16.6%)	–	–	1 (33.3%)	1 (50%)
**Antibiotics**	–	–	–	2 (18.2%)	1 (25%)	–	–
**Hepatics and pancreatics**	–	–	–	3 (27.3%)	1 (25%)	–	1 (50%)
**Hepatics and Hidrocortisone pre-IFX^b^**	–	–	–	1 (9.1%)	–	–	–
**Other**	2 (40%)	1 (100%)	2 (16.6%)	4 (36.4%)	2 (50%)	1 (33.3%)	2 (100%)

^a^CD, Crohn’s disease; ^b^IFX, infliximab.

### Isolation of Leukocytes and Detection of AnxA1 in the Blood of CD Patients

Blood from healthy donors (*n* = 5) and CD patients treated with IFX (*n* = 23) or not (*n* = 1) was used to isolate the following:

Plasma AnxA1: AnxA1 was detected in plasma samples using an ELISA kit (MyBioSource, San Diego, CA, USA).

Leukocytes: NH_4_Cl (0.13 M) was added to the remaining cell fraction to lysate erythrocytes. Pellets were fixed in 1% paraformaldehyde (PFA) and incubated with anti-FPR1 (PE, R&D Systems, Minneapolis, MN, USA) and anti-FPR2 (FITC, Bioss, Woburn, MA, USA) antibodies. Readings were conducted using a BD Accuri Flow Cytometer (BD Biosciences, Franklin Lakes, NJ, USA) to acquire 10,000 events/sample. Positive populations were determined by labeling for each antibody separately.

### Detection of AnxA1, FPR1 and FPR2 in CD Biopsies

Paraffin-embedded colon biopsies from CD untreated (*n* = 4) and treated positive (*n* = 3) or negative (*n* = 2) responders to IFX were permeabilized (0.01% Triton), retrieved (sodium citrate buffer), and blocked (20% fetal bovine serum, FBS). Samples were incubated with mouse anti-FPR1 (1:25, clone 5F1; BD Biosciences), anti-FPR2 (1:10, clone 2D8; Sigma-Aldrich, St. Louis, MO, USA), or anti-AnxA1 (1:50, clone 1B, 10 μg/ml). Incubation with 20% FBS provided the negative control. After incubation with anti-mouse Alexa Fluor 488 secondary antibody (1:200; Thermo Fisher, Waltham, MA, USA) and DAPI, the slides were mounted and five regions of interest (ROIs) per slide were photographed on a confocal microscope (Zeiss LSM800). Before acquiring images, the settings for gain, offset, and exposure time were adjusted based on the reaction control and standardized for each ROI from the stained samples. Acquired composite images (.czi format) were imported to Fiji (ImageJ Software, Bethesda, MD, USA) and split into blue and green channels. For densitometric analysis, the green channel (Alexa Fluor 488) was selected and modified to be displayed with a gray filter. Background pixel averages were then subtracted from the image pixels of interest to correct uneven illumination with the aid of the “Process > Math > Subtract” process. Fluorescence measures were performed manually by the selection of positive regions; average values were expressed in arbitrary units.

### Ethics Statement and Animals

C57BL6 wild-type (WT) or AnxA1-null mice (AnxA1^−/−^), males, 8–10 weeks old, were used to perform colitis. WT C57BL6, males, 16–18 weeks old, were used to provide intestinal immune cells to *ex vivo* experiments. Mice were obtained from the Federal University of São Paulo Animal House (Brazil), kept in 12:12-h light/dark cycle, and provided with food and water *ad libitum*. Experiments were performed in accordance with the Brazilian laws of protection and approved by the Committee on Ethics of Animal Experiments from the University of São Paulo.

### Colitis Model and Clinical Analysis

Fresh 2% dextran sodium sulfate (DSS; weight/volume, 40 kDa; Dextran Products Limited, Scarborough, Ontario, Canada) was added to the drinking water of WT and AnxA1^−/−^ mice (day 0) and replenished every other day up to day 6 (26). Control and non-treated DSS mice received vehicle (sterile saline 0.9% + DMSO 0.5%) intraperitoneally (i.p.) on days 0–9. DSS-treated groups received i.p. IFX (1 mg/kg; Remicade^®^ Janssen-Cilag, Buenos Aires, Argentina) on day 1 and/or FPR antagonist Boc-2 (10 μg/kg; N-t-BOC-MET-LEU-PHE, MP Biomedicals, Irvine, CA, USA) on days 0–9. Body weight, diarrhea, and rectal bleeding were scored daily to provide the Disease Activity Index (DAI). On day 10, mice were euthanized by overexposure to nasal anaesthesia [isoflurane; 2-chloro-2-(difluoromethoxy)-1,1,1-trifluoro-ethane].

### Dosage of Tissue MMP-9

Samples of medium colon were homogenized in RIPA buffer containing a protease inhibitor (1:100; Thermo Fisher) in an Ultra-Turrax homogenizer (T10-Basic-IKA). After 20 min on ice, the tissue debris were removed and matrix metalloproteinase 9 (MMP-9) was detected by ELISA (R&D Systems).

### Histological Analysis *In Vivo*

Distal colons were fixed in 4% buffered PFA, dehydrated, and embedded in paraffin. Samples were analyzed using high-power objectives on the Imager.A2 Zeiss microscope (Zeiss, Oberkochen, Germany).

#### Histopathology

Histological grading was based on a previous report (8). The following features were analyzed by a blinded histologist: changes on crypts and histoarchitecture, edema, ulceration and immune cells at the epithelium, lamina propria, or submucosa. Grades of 0, 1, 2, 3, and 4 were respectively attributed to normal, mild, mild–moderate, moderate–severe, and severe conditions. Results were expressed as the mean of total grading.

#### Immunohistochemistry

After permeabilization (Triton 0.01%) and antigen retrieval (sodium citrate buffer, 10 mM, pH 6.0), peroxidase was inactivated with 3% hydrogen peroxide. Samples were blocked with 10% Tris-buffered saline–bovine serum albumin and incubated with the anti-AnxA1 antibody (1:500; Thermo Fisher). The reaction control was incubated with a blocking solution. The slides were finalized with an anti-rabbit horseradish peroxidase (HRP) antibody (Abcam, Cambridge, UK), 3,3-diaminobenzidine (DAB; Thermo Fisher), and hematoxylin counterstaining.

#### Immunofluorescence

Permeabilized samples were retrieved in sodium citrate buffer and blocked with 20% FBS. Antibodies were incubated overnight (4°C): polyclonal mouse anti-β-actin (1:200) and rabbit anti-villin (1:50; Abcam). After secondary antibodies (anti-mouse DyLight 549 and anti-rabbit FITC, 1:200; Vector Laboratories, Burlingame, CA, USA), the nuclei were stained with DAPI.

### Isolation of Leukocytes From Lamina Propria and Flow Cytometry

Leukocytes from proximal colonic lamina propria were isolated after washes with 2 mM EDTA and digestion with collagenase V from *Clostridium histolyticum* (1 mg/ml; Sigma-Aldrich) (26). The cells were washed through 40-μm strainers (Corning, Corning, NY, USA) and stained with CD4 (FITC) and CD25 (APC) (1:100; BD Biosciences). Positive populations were determined by labeling with single antibodies. A minimum of 10,000 events per sample were acquired on a BD Accuri Flow Cytometer. The results were expressed as percentages of positive cells normalized by controls from each experiment.

### Isolation of Colonic Lamina Propria Leukocytes and *Ex Vivo* Treatments

After euthanasia by overexposure to isoflurane, colons from C57BL6 mice were opened longitudinally and washed with supplemented phosphate-buffered saline (10,000 μg/ml penicillin/streptomycin and 50 μg/ml gentamycin). Under a sterile hood, the tissues were fragmented, washed in Hank’s salt solution buffer without calcium/magnesium for 20 min (twice), and digested with collagenases from *C. histolyticum* (types II and IV, 0.5 mg/ml; Gibco, Waltham, MA, USA). The digested tissue was washed twice through 40-μm strainers (Corning) and the pellets were counted and resuspended at the Roswell Park Memorial Institute (RPMI + 1% FBS). Cells were seeded in a 96-well plate (2 × 10^5^/well) and treated with 200 ng/ml lipopolysaccharide (LPS; Sigma-Aldrich) 30 min before IFX (0.1, 1.0, or 10.0 μg/ml). Controls were untreated or treated with those IFX doses. After 24 h, the supernatants were collected to dose secreted AnxA1 (MyBioSource) according to the manufacturer’s instructions.

### Statistical Analysis

To determine the parametric or non-parametric distributions, we used the Kolmogorov–Smirnov test. ANOVA followed by Tukey’s was performed for parametric tests, and Kruskal–Wallis followed by Dunn’s post-test was performed for non-parametric tests. To compare the two groups, we applied unpaired (single measures) or paired *t*-test (repeated measures). Pearson’s correlation was performed for correlation analysis, providing the correlation coefficient *r* and the coefficient of determination *R* ^2^. Probabilities with *p* < 0.05 were considered significant. The results were expressed as the mean ± standard deviation (SD; human samples, individual variabilities) or standard error of the mean (SEM; *in vivo*/*ex vivo* assays, group variability). All statistical assessments were conducted using GraphPad Prism^®^ software, version 9.1.2 (San Diego, CA, USA).

## Results

### AnxA1 and FPR1 Are Differentially Expressed in the Colon of CD Patients Responsive to IFX

Recently, it has been suggested that differential expressions of AnxA1 might allow the identification of disease severity patterns in IBD (14, 17). Thus, we proposed screening the expression patterns of AnxA1 and its receptors, FPR1 and FPR2, in CD biopsies of colons from untreated and IFX-treated patients who either went into remission or did not. Information about the healthy volunteers and CD patients are listed in **Table 1**.

**Figure 1:**
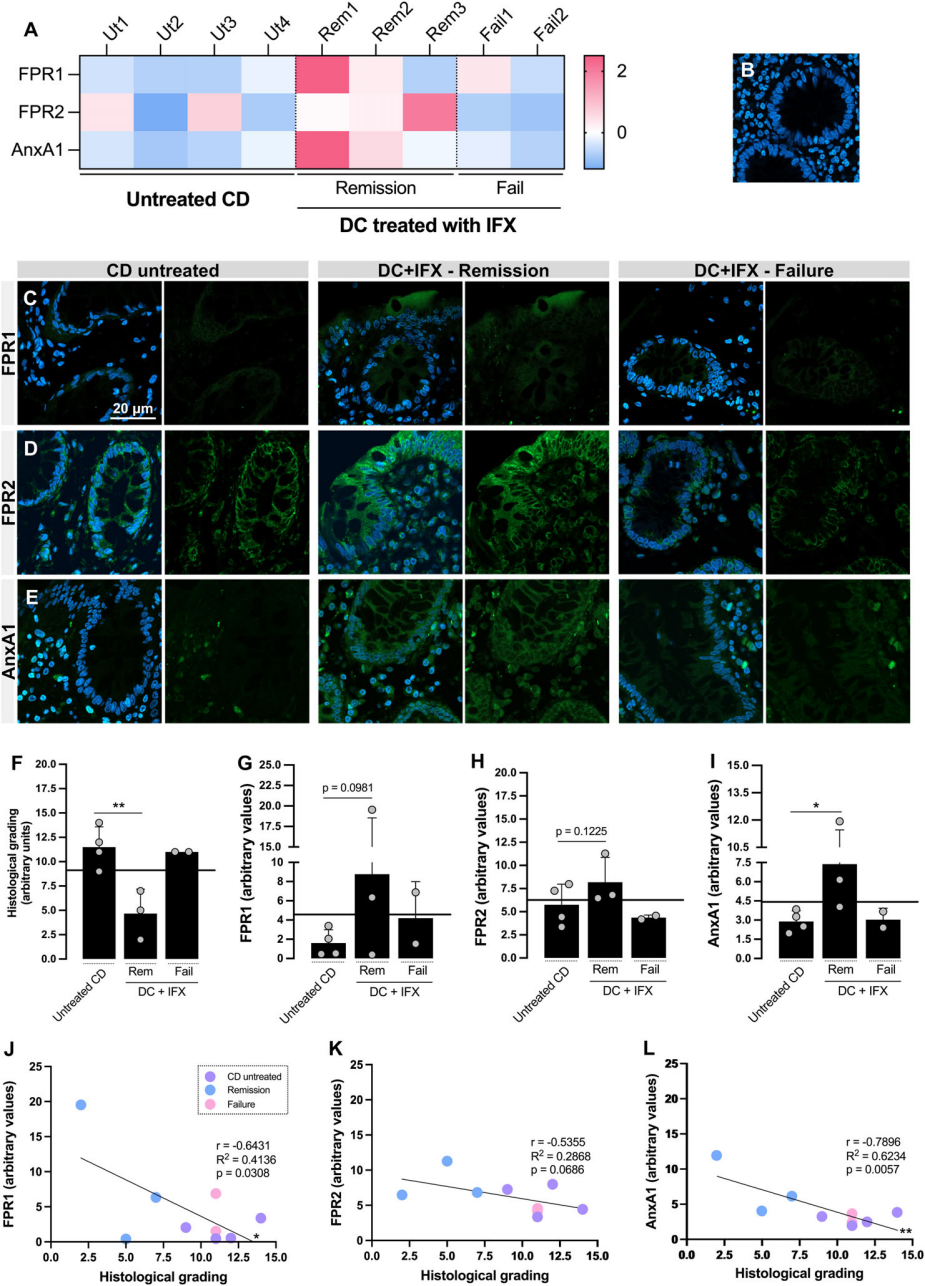
Formyl peptide receptor 1 (FPR1), FPR2, and annexin A1 (AnxA1) are differentially expressed in the colon of Crohn’s disease (CD) responders to infliximab (IFX) and correlate with histological homeostasis. **(A)** Heatmap based on the *Z*-scores of FPR1, FPR2, and AnxA1 expression values in the colon. **(B–E)** Confocal imaging: negative control of reaction **(B)**, FPR1 **(C)**, FPR2 **(D)**, and AnxA1 **(E)** tissue staining. Embedding, paraffin; sections, 3 μm. *Bar*, 20 μm. **(F)** Histological grading, calculated based on **Supplementary Table 1**. **(G–I)** Densitometric analysis of fluorescence: FPR1 **(G)**, FPR2 **(H)**, and AnxA1 **(I)**. **(J–L)** Correlation analysis between histological grading of biopsies and fluorescence intensity of staining: FPR1 **(J)**, FPR2 **(K)**, and AnxA1 **(L)**. *p < 0.05, **p < 0.01. *n* = 4 (CD untreated); *n* = 3 (Remission); *n* = 2 (Failure). Results are expressed as the mean ± SD.

Hematoxylin/eosin-stained colon biopsies revealed ulcers, crypt alterations/abscesses, and prominent inflammatory infiltrates in untreated and IFX Failure patients (**Supplementary Figures 1A, C**). Based on the grading system (**Supplementary Table 1**), we confirmed lower histological damage for remittent (**Supplementary Figure 1B** and **Figure 1F**) rather than untreated CD individuals.

Subsequently, FPR1, FPR2, and AnxA1 were detected on the colon biopsies using confocal microscopy (**Figure 1**). For those three markers, the *Z*-scores were above the population mean for the IFX-induced remission group, contrasting with the lower levels in the untreated and IFX Failure groups (**Figure 1A**). Despite the increased fluorescence detected for FPR1 (**Figures 1C, G**) and FPR2 (**Figures 1D, H**) in remittent patients, only AnxA1 was significantly higher than that in the untreated group (**Figures 1E, I**). Epithelial cells from barrier, crypts, and leukocytes were the major sources of FPR1, FPR2, and AnxA1. Furthermore, colonic FPR1 and AnxA1 expressions presented a strong negative correlation with the histological grading (**Figures 1J, L**), which means that the decrease of these markers is associated with more tissue damage. For FPR2, a moderate correlation was detected (*r* = −0.5355), but this was not enough to characterize a significant association (**Figure 1K**). Based on this, we presume that the upregulation of colonic FPR1 and AnxA1 in CD positive responders to IFX provides a marker of differential expression for therapy efficacy.

Using the patients’ medical histories, we calculated the CDAI (**Supplementary Table 2**). Despite the different intensities of disease activity among patients, no patterns for circulating FPR1, FPR2 (leukocytes), or AnxA1 (plasma) were observed for the healthy, remission, or failure groups (**Supplementary Figure 2A**). As expected, CDAI was significantly higher in IFX-unresponsive patients than in remission patients (**Supplementary Figure 2B**). Furthermore, FPR1, FPR2, and AnxA1 in blood did not correlate with CDAI, indicating that patients’ circulating levels might not be reliable biomarkers for remission or failure after IFX treatment (**Supplementary Figures 2C–H**).

### FPR Signaling Complements the Beneficial Effects of IFX on Colitis Symptomatology and Tissue Damage

**Figure 2:**
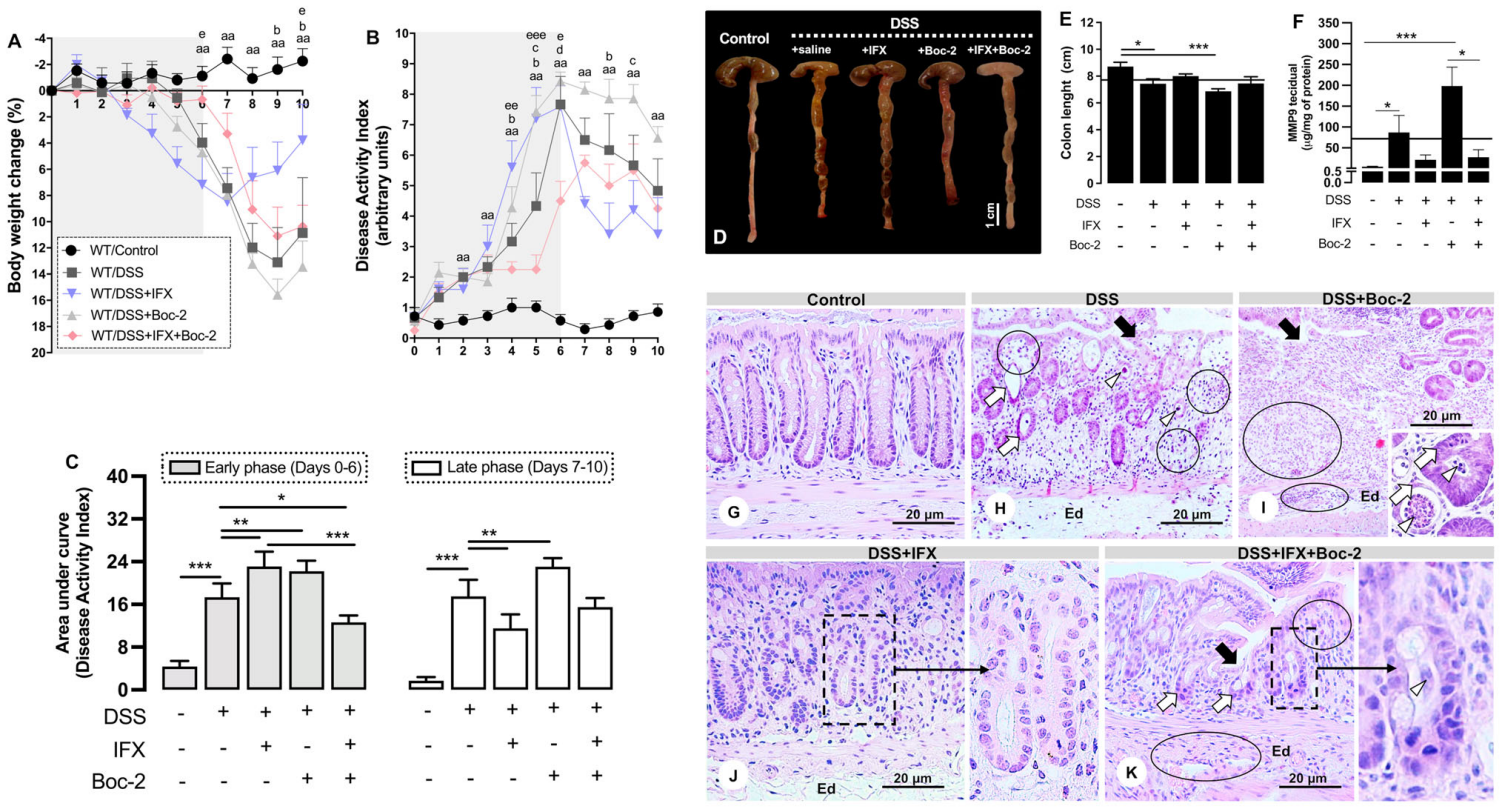
Formyl peptide receptor (FPR) blockade impairs clinical and histological improvements induced by infliximab (IFX) during colitis. **(A)** Loss of body weight. **(B)** Disease Activity Index (DAI). **(C)** Area under the curve from DAI, early and late phases. **(D, E)** Colon length and anatomic changes. **(F)** Colonic MMP-9 levels. Mean of all samples (*continuous lines*). *Bar*, 1 cm. ^aa^ *p* < 0.01 (DSS *vs.* Control); ^b^ *p* < 0.05 (DSS+IFX *vs.* DSS); ^c^ *p* < 0.05 (DSS+Boc-2 *vs.* DSS); ^d^ *p* < 0.05 (DSS+IFX+Boc-2 *vs.* DSS); ^e^ *p* < 0.05, ^ee^ *p* < 0.01, ^eee^ *p* < 0.001 (DSS+IFX+Boc-2 *vs.* DSS+IFX). *p < 0.05, **p < 0.01, ***p < 0.001. *n* = 4–6 mice/group. Results are expressed as the mean ± SEM. **(G–K)** Histopathology in wild-type (WT) groups. Ulcer (*black arrows*), altered crypts (*white arrows*), crypt abscesses (*white arrowheads*), edema (*Ed*), and inflammatory infiltrate (*circles*). Staining, hematoxylin–eosin; embedding, paraffin; sections, 3 μm. *Bars*, 20 μm.

After detecting the correlations between high expressions of FPR1/AnxA1 and histological recovery in CD, we wondered how the modulation of these markers would impact resolution after IFX. We have previously demonstrated the failure of IFX in treating acute colitis using AnxA1-null Balb/c mice (8). To explore the mechanisms involved in the efficacy of IFX, we performed DSS-induced colitis in WT and AnxA1^−/−^ C57BL6 mice and followed up to the late phase of the disease (**Figure 2** and **Supplementary Figures 3, 4**). As expected, colitis induced significant weight loss and increased the DAI in both WT (**Figures 2A–C**) and AnxA1^−/−^ strains (**Supplementary Figures 4A, B**). Anatomic and microscopic alterations were also produced by DSS, including colon shortening, MMP-9 increase, ulcers, altered crypt, crypt abscesses, vacuolar hydropic degeneration, submucosal edema, and mucosal/submucosal massive inflammatory infiltrates (**Figures 2D–H** and **Supplementary Figures 4E–I**). IFX attenuated body weight loss (days 9 and 10; **Figure 2A**) and DAI during the late phase of the disease (day 8; **Figures 2B, C**) in WT mice. Except for some remaining infiltrated leukocytes and submucosal edema, the colonic histological architecture was protected by IFX (**Figure 2J**). These improvements were not only absent in the AnxA1^−/−^ group upon IFX treatment (**Supplementary Figures 4A, B, J**), but anti-TNF-α seemed to be harmful without endogenous AnxA1 as well: a 50% mortality rate was recorded (**Supplementary Figure 4C**). AnxA1-deficient mice had significant colon shortening (**Supplementary Figures 4E, F**) and a 200- to 300-fold increase in tissue MMP-9 with or without IFX treatment (**Supplementary Figure 4G**). Moreover, AnxA1^−/−^/DSS+IFX mice had less intestinal T regulatory lymphocytes than did the mice in the respective WT group and the untreated group (AnxA1^−/−^/DSS), reaffirming the relevance of AnxA1 to the downregulation of inflammation even when TNF-α is being blocked by IFX (**Supplementary Figure 4D**).

For the next step, we blocked the AnxA1 receptors, FPRs, to assess their potential involvement in the effects of IFX. The FPR blockade, by itself, confirmed their well-known role in wound healing (15, 27). FPR neutralization was detrimental to the clinical parameters in both early and late phases of disease (**Figures 2A–C**), intensified colon shortening (**Figures 2D, E**), and increased MMP-9 (**Figure 2F**). Microscopically, the FPR blockade with Boc-2 worsened the DSS damage, especially with regard to epithelial/glandular loss and infiltrated leukocytes (**Figure 2I**). When combined with IFX, blocking FPRs was detrimental to the clinical conditions in the late phase as the beneficial effects of IFX on body weight and DAI were lost (days 9 and 10 and day 8 in **Figures 2A, B**, respectively) (**Figure 2C**). Although the colon length and MMP-9 from IFX-treated mice were not affected by Boc-2 (**Figures 2D, E**), the cecum very consistently assumed a distorted morphology and paleness for this group (**Figure 2D**).

Finally, the histological parameters were analyzed. IFX-mediated preservation of the colonic histoarchitecture was partially impaired by an FPR blockade, with the presence of immune cells in the mucosa and punctual but persistent ulcerations (**Figure 2K**). The clearest histological alteration in this group was poor crypt recovery (details in **Figure 2K**). For this reason, we proceeded to explore the effects of halting the signaling through FPRs for the regeneration of crypts after IFX treatment.

### Blocking FPRs Compromises the Effects of IFX on Crypt Regeneration

**Figure 3:**
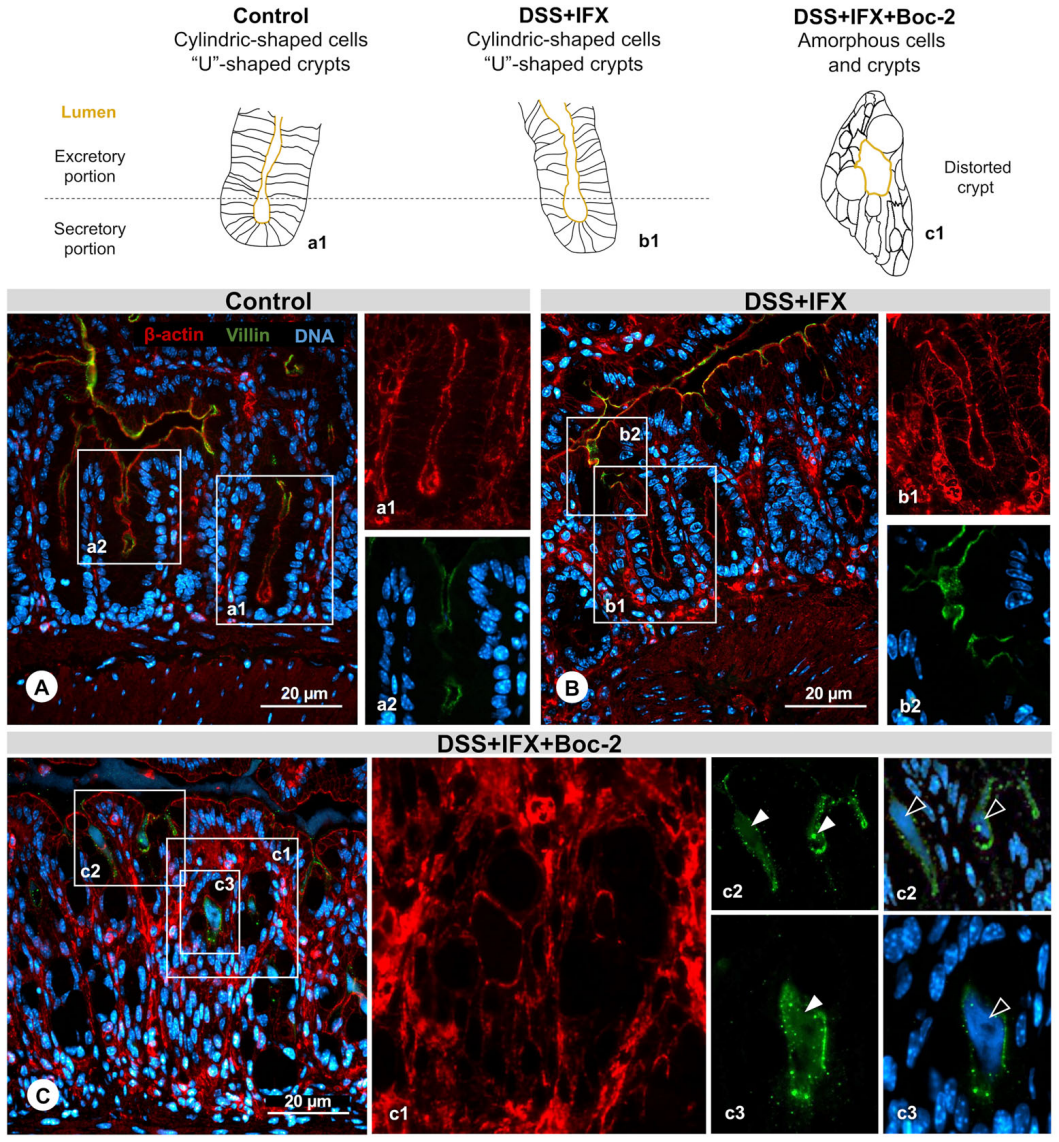
Crypt regeneration mediated by infliximab (IFX) requires formyl peptide receptor (FPR) signaling. **(A)** Control: cylindrical-shaped cells forming a U-shaped crypt revealed by β-actin staining (*a1*, *red*). Presence of villin (*a2*, *green*) in the inner surface from the excretory part of the crypt lumen. Representation of a normal structure. **(B)** DSS+IFX: preserved crypt morphology resembling the control condition (*b1* and *b2*). **(C)** DSS+IFX+Boc-2: defects in crypt closure (*c1*); presence of villin (*c2* and *c3*, *white arrowheads*); and DNA residue (*c2* and *c3*, *black arrowheads*) in the crypt lumen. *n* = 4–6 mice/group. Embedding, paraffin; sections, 3 μm. Bars, 20 μm.

The relevance of FPRs for crypt regeneration was confirmed by structural protein immunostaining (**Figure 3**). In control mice, β-actin revealed the cylindric shape of enterocytes (**Figure 3A**, a1) with villin expressed on the apical surfaces of cells (**Figure 3A**, a2). A similar pattern was observed on crypts from DSS+IFX mice (**Figure 3B**, b1 and b2). In both groups, it is possible to identify the secretory and excretory portions from the mucosal glands, forming a U-shaped structure with a centered vertical lumen that opens to the intestinal wall, where the secretions produced by crypts are liberated.

Upon FPR inactivation, with or without IFX treatment, crypt cells assumed a deformed morphology lacking cylindric limits. The elongated shape from crypts gave place to an amorphous structure, many times with the lumen turned into a round form that was no longer connected with the epithelium surface (**Figure 3C**, c1). In this space, we observed residues of the shed villin (**Figure 3C**, c2 and c3, green) that co-localized with DNA (**Figure 3C**, c2 and c3, blue). FPRs were associated with epithelial repair, migration, and wound healing before, but our results suggest that these roles are also required for the intestinal barrier homeostasis mediated by IFX. Considering that AnxA1 is an important FPR agonist and a pivotal mediator of tissue repair in IBD, our final step was to assess the possible kinds of interplay among AnxA1 expression, FPR activation, and IFX efficacy.

### IFX Induces AnxA1 Expression and Secretion by Tissue Leukocytes During Inflammation

**Figure 4:**
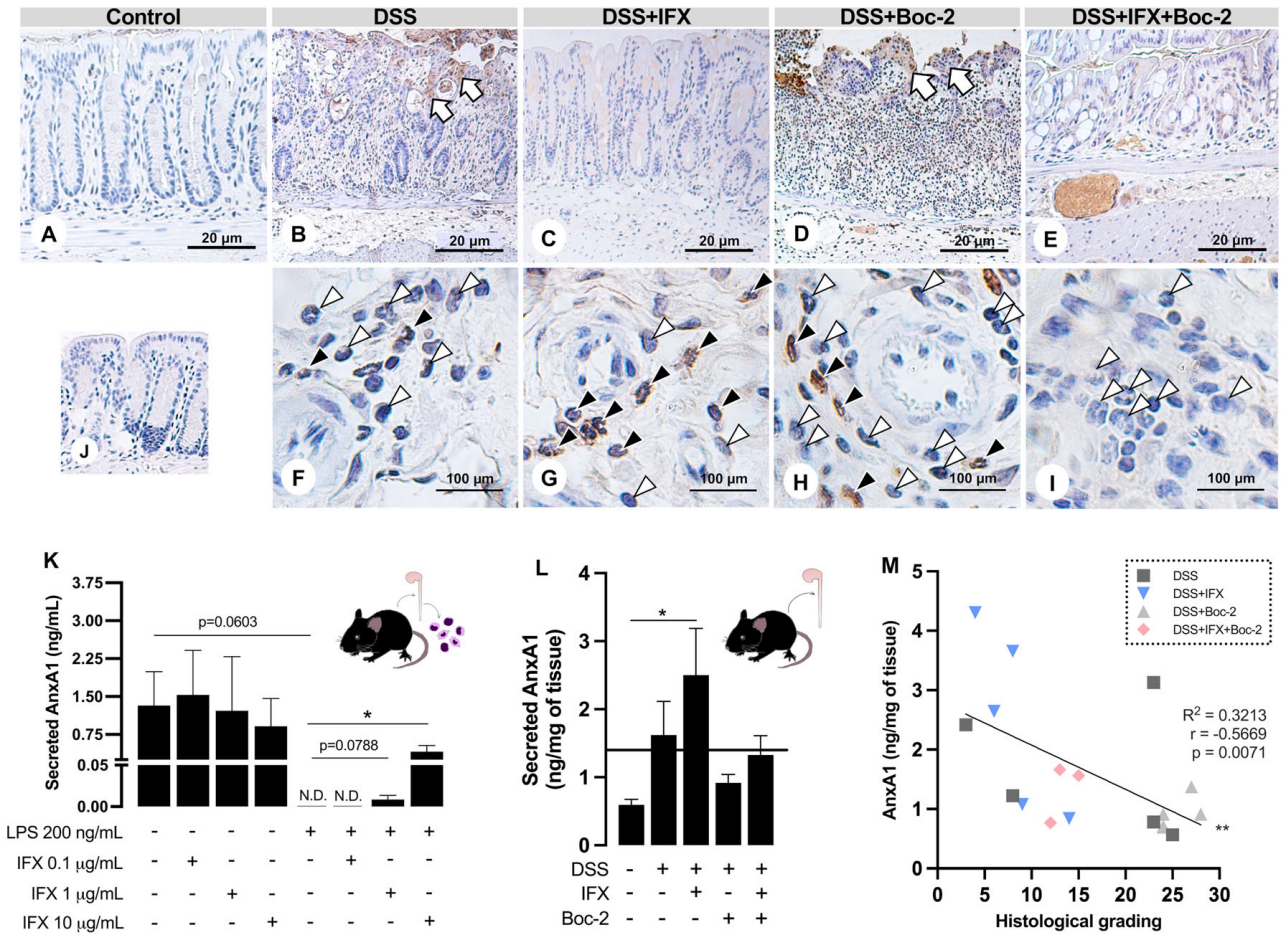
Infliximab (IFX) induces annexin A1 (AnxA1) expression and secretion by infiltrated leukocytes during inflammation. **(A–E)** AnxA1 expression in the epithelial barrier from wild-type (WT) mice colon. AnxA1 concentrated on the wound bed (*white arrows*). **(F–I)** Immunostaining for AnxA1 in submucosal leukocytes. Tissue leukocytes positive (*black arrowheads*) or negative (*white arrowheads*) for AnxA1 staining. **(J)** Reaction control. Counterstaining, hematoxylin; embedding, paraffin; sections, 3 μm. *Bars*, 20 μm **(A–E)** and 100 μm **(F–I)**. **(K)** AnxA1 secreted by lamina propria leukocytes cultured *ex vivo*. **(L)** AnxA1 secreted by colon explants in culture. **(M)** Moderate negative correlation between AnxA1 secreted into the tissue and histological grading. *N.D.*, non-detected. *p < 0.05, **p < 0.01. *n* = 3–4 mice/group **(K)**; *n* = 4–6 mice/group **(L)**. Results are expressed as the mean ± SEM.

Intracellular and FPR-binding AnxA1 mediate inflammation resolution and tissue repair on experimental colitis (8, 28). In our model, we assessed the expression patterns for endogenous AnxA1 in the colon (**Figure 4**). The reaction control confirmed specificity (**Figure 4J**).

In the control epithelium, AnxA1 was weakly detected (**Figure 4A**). Colitis upregulated AnxA1 in the damaged epithelium from the DSS and DSS+Boc-2 groups (**Figures 4B, D**). Mice treated with IFX, with or without FPR signaling, presented epithelial AnxA1 similar to the control group (**Figures 4C, E**). In turn, AnxA1 was weakly detected in tissue leukocytes in non-treated colitis (**Figure 4F**). After IFX, a high number of infiltrated AnxA1-positive immune cells were observed (**Figure 4G**). AnxA1 was also detected in leukocytes under Boc-2 (**Figure 4H**). Interestingly, in mice treated with IFX and Boc-2, most tissue leukocytes were negative for AnxA1 (**Figure 4I**).

Because the intracellular expression of AnxA1 was upregulated in tissue leukocytes upon IFX, we hypothesized that transmigrated immune cells could be important sources of AnxA1 for the inflamed tissue treated with this anti-TNF-α. Using LPS *in vitro* assays, we simulated an inflammatory response in immune cells isolated from the colon of naive mice. When treated with increasing concentrations of IFX (0.1, 1.0, and 10 µg/ml), we detected a dose-dependent augmentation in AnxA1 secretion to the milieu (**Figure 4K**). This phenomenon was reproduced in the DSS-induced colitis treated with IFX, as colon explant supernatants had increased levels of AnxA1 compared to the control group (**Figure 4L**). Finally, we observed a moderate negative correlation between the secreted AnxA1 and the grading for histological damage of the colon (**Figure 4M**). These results suggest that IFX induces AnxA1 expression and secretion by tissue leukocytes, and this mechanism is linked to tissue repair.

## Discussion

Previously, we reported the relevance of AnxA1 in the efficacy of IFX during acute colitis in female Balb/c mice (8). Here, we corroborate it using mice of different strain and sex and focusing on the late phase of disease. On biopsies from CD patients, we further confirm that IFX modulates AnxA1 in positive responders. Moreover, we describe FPR1 as a potential marker of tissue homeostasis after IFX treatment. Our results suggest that IFX stimulates activated intestinal leukocytes to express and secrete AnxA1 to the inflamed milieu, where it is free to bind to FPRs and promote tissue repair (29).

Although biologicals are the most successful drug class at inducing remission among the therapies classically used to treat IBD (30), they still burden patients with heterogeneous outcomes, including hypersensitivity, allergies, lack of responsiveness, or progressive loss of efficacy over the lifetime (31). Even clinically remittent patients may present remaining mucosal inflammation associated with severe mucosal disease activity (32). In response to this, the follow-up protocol for treated patients proposes the assessment of histological remission as a more reliable tool to predict a sustained and steroid-free clinical remission, with lower need for hospitalization and surgery (33). Herein, we observed a marked expression of AnxA1 in the colon of CD patients who underwent remission after IFX. For AnxA1 and FPR1, high tissue levels correlated with lower histological damage. In a general manner, the literature on IBD suggests that AnxA1 secretion is evoked during the active disease. Failures in this mechanism contribute to a more severe and prolonged disease (26, 34). Similarly, therapeutic interventions have a better outcome when capable of increasing AnxA1 levels (8, 14). In the inflamed site, AnxA1 binding to FPRs mediates the resolution of inflammation and mucosal homeostasis (16, 28). The data we present here support the mechanisms described above. Moreover, they suggest that tissue FPR1 and AnxA1 could constitute useful tools for following up the disease activity and the efficacy of IFX in CD patients.

**Figure 5:**
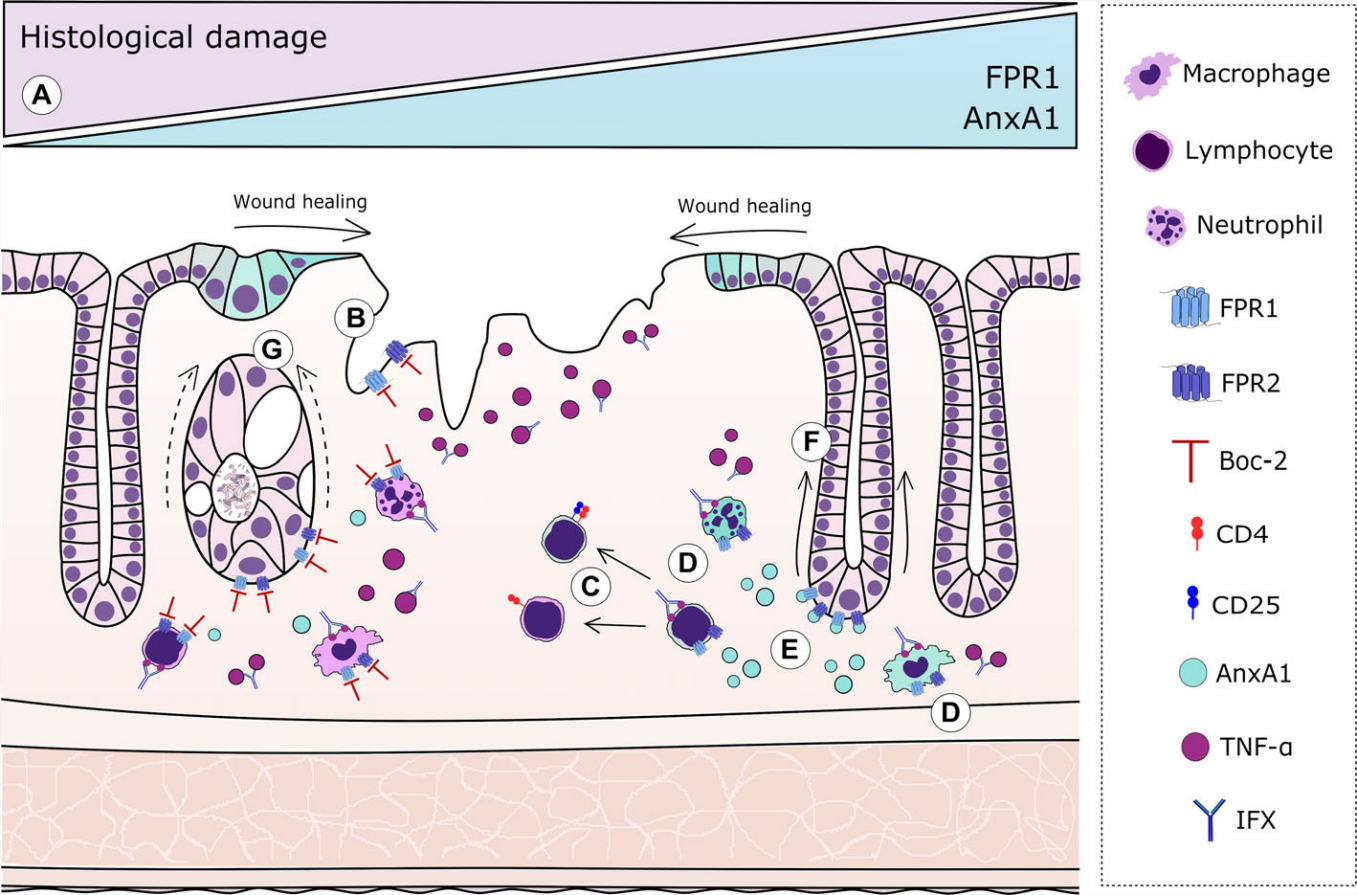
Graphical representation of the resolutive effects of the annexin A1 (AnxA1)–formyl peptide receptor (FPR) axis complementary to infliximab (IFX) mechanisms. *In humans*: **(A)** Increased expressions of FPR1 and AnxA1 in colonic epithelial cells and lamina propria leukocytes correlate with histological remission after IFX. *In mice*: **(B)** AnxA1 expression is evoked in epithelial cells from damaged areas, but blockage of FPRs impairs proper re-epithelization. **(C)** In tissue T lymphocytes, the absence of endogenous AnxA1 skews cells to a more pro-inflammatory profile, resulting in the decreased number of regulatory T cells (Tregs). **(D)** Immune cells transmigrated to the intestinal tissue are important sources of AnxA1 after IFX. **(E)** IFX induces AnxA1 secretion to the inflammatory milieu, which correlates with histological recovery. **(F)** In IFX-treated colitis, proper signaling of FPRs is crucial to adequate crypt regeneration. **(G)** Blockade of FPRs impairs the ability of IFX to protect glandular cells and restore the characteristic U-shape of crypts, producing amorphous structures after loss of basal–apical orientation and β-actin/villin distribution.

Mucosal inflammation begins with the opening of the epithelial barrier after injury, which allows the translocation of microbial agents. Once the commensal microbiota is detected by the immune cells that patrol the tissue, an inflammatory response is assembled to contain the spreading of bacteria to the blood and other tissues. However, an unbalanced inflammation feed-forwards tissue damage and increases bacterial translocation (35). Upon injury or death, column-shaped intestinal epithelial cells can lose contact with each other, assuming a flat morphology. The organism responds by triggering a proliferation of basal crypt cells and epithelial migration, promoting wound closure (36). By binding FPRs, full-length AnxA1 and its peptide, Ac2-26, stimulate the migration of epithelial cells and wound healing (15, 16). Ac2-26 binding to FPR1 induces oxidative inactivation of phosphatase and tensin homolog protein and protein tyrosine phosphatase-PEST, phosphorylating focal adhesion kinase and paxillin, consequently stimulating cells to migrate (15). Additionally, endogenous AnxA1 acts as an anchor between cytoplasmic β-actin and the plasma membrane, whereas the AnxA1–FPR2 axis inhibits RhoA GTPase activity, stabilizing the cytoskeleton (37). Our results confirmed that the lack of endogenous AnxA1 is very detrimental to the progression of experimental colitis: AnxA1^−/−^ mice had much worse clinical and histological outcomes, fewer intestinal regulatory T cell (Treg) counts, and a 25% mortality rate compared to WT mice. Preventing AnxA1 and other agonists from binding with FPRs *in vivo* was almost equally harmful as all clinical parameters were impaired and tissue damage was remarkable, especially considering crypt and epithelial loss. Interestingly, treatment with IFX did not compensate for the absence of endogenous AnxA1 or FPR signaling. The blockade of the AnxA1–FPR axis impaired crypt restitution, whereas a lack of AnxA1 impacted the ability of IFX to promote wound healing and to rebalance the immune response. Based on these data, we propose that protection of the epithelial barrier with IFX must be complemented by the downstream pathways resulting from the binding of AnxA1 (and other endogenous agonists) to FPRs (**Figure 5**).

It is well known that the use of IFX on chronic diseases modulates cellular pathways downstream of TNF receptors (TNFRs) by blocking soluble and transmembrane TNF-α (38, 39). Considering the complexity of the mechanisms evoked by IFX and the heterogeneous responses it produces, other endogenous players are likely to be involved in successful outcomes. Herein, we suggest that AnxA1 expression might be one mechanism induced by IFX, which may, in turn, regulate its effects at some level. This notion is supported by the modulation of intracellular and secreted AnxA1 after IFX treatment in colitic mice. In colonic mucosal and submucosal leukocytes activated by inflammatory mediators, IFX evoked AnxA1 expression. The same group had increased secretion of AnxA1, which was most likely being provided by those leukocytes. In line with this, increased levels of AnxA1 were secreted as higher doses of IFX were added to LPS-stimulated intestinal leukocytes *ex vivo*. These results seem to be translatable to the human condition because we also observed increased AnxA1 in biopsies from CD patients in association with histological remission. Indeed, the lack of TNF-α signaling through TNFR1 in mice during colitis favors tissue AnxA1 expression and increases the frequency of CD4^+^ and CD8^+^ T lymphocytes positive for AnxA1 (26). These data indicate that intestinal immune cells are the major sources of secreted AnxA1; this could be one resolutive mechanism of IFX that was not previously described. Interestingly, the Boc-2 groups had less secreted AnxA1 with or without IFX. This suggests a more complex mechanism of action for IFX, in which proper FPR signaling is required to induce AnxA1 secretion by leukocytes and, in turn, to mediate tissue repair and wound healing.

Obviously, FPR agonists other than AnxA1 could be involved in the efficacy of IFX. In the inflammatory milieu, FPR ligands can be found among endogenous metabolites, peptides, and damage- and pathogen-associated molecular patterns (DAMPs and PAMPs, respectively) (40). These include *N*-formyl-peptides from mitochondrial damage (41), the antimicrobial peptide LL-37 and lipoxin A4 (22). It should be noted that the bacterial-derived peptide formyl-methyl-leucine-phenylalanine (fMLP) can be present at high concentrations in the wounded intestinal tissue (42) and binds FPR1 with a strong affinity, triggering healing pathways on epithelial cells (20, 43). Despite that, this work addresses AnxA1 participation because of its pivotal roles in IBD. Our choice was also supported by the increased AnxA1 secretion upon IFX in experimental colitis and its correlation with tissue recovery. It is also important to point out that, although we argue that AnxA1 and FPRs contribute to the efficacy of IFX, it is possible that they might be involved in the effects of other therapies as well. We do not claim that this modulation is exclusive to IFX, but that it obtains for this specific therapy. We hope that our study encourages others to explore therapies that might be complemented by AnxA1–FPR, elucidating possible mechanisms of therapeutic failure in IBD. We believe that the data we presented here may contribute to the identification of poor responsiveness cases, leading to long-term remission based on the pivotal homeostatic roles of AnxA1 through FPRs.

## Data Availability Statement

The original contributions presented in the study are included in the article/**Supplementary Material**. Further inquiries can be directed to the corresponding author.

## Ethics Statement

The studies involving human participants were reviewed and approved by the boards on ethics from Santa Casa and University of São Paulo. The patients/participants provided their written informed consent to participate in this study. The animal study was reviewed and approved by the Committee on Ethics of Animal Experiments, University of São Paulo.

## Author Contributions

MS and SF conceived the hypothesis, designed the study, interpreted the data, and prepared the manuscript. MS, GR, MB, SS, and RL designed and carried out the experiments. MS, MQ, and AV participated in patient approach and collecting samples and medical history. SO, MP, and SF provided scientific supervision, infrastructure, and essential tools to perform experiments. All authors contributed to the article and approved the submitted version.

## Funding

This work was supported by Fundação de Amparo a Pesquisa do Estado de São Paulo, Brazil (grant nos. FAPESP 2014/07328-4, 2016/19682-2 and 2019/02806-9).

## Conflict of Interest

The authors declare that the research was conducted in the absence of any commercial or financial relationships that could be construed as a potential conflict of interest.

## Publisher’s Note

All claims expressed in this article are solely those of the authors and do not necessarily represent those of their affiliated organizations, or those of the publisher, the editors and the reviewers. Any product that may be evaluated in this article, or claim that may be made by its manufacturer, is not guaranteed or endorsed by the publisher.
