# Supplementary material for: Formyl Peptide Receptors and Annexin A1: Complementary Mechanisms to Infliximab in Murine Experimental Colitis and Crohn’s Disease
Source: Front Immunol. 2021 Sep 17;12:714138. doi: 10.3389/fimmu.2021.714138 (PMC8484756; doi:10.3389/fimmu.2021.714138)
Supplement: Supplementary file 1 [file DataSheet_1.docx]

**Frontiers In Immunology**

**Supplementary information**

**Formyl-peptide receptors and annexin A1:**

**complementary mechanisms to infliximab in**

**murine experimental colitis and Crohn’s disease**

M. de Paula-Silva; G.H.O. da Rocha; M.F. Broering; M.L. Queiroz; S. Sandri; R.A. Loiola; S.M. Oliani; A. Vieira; M. Perretti; S.H.P. Farsky

**
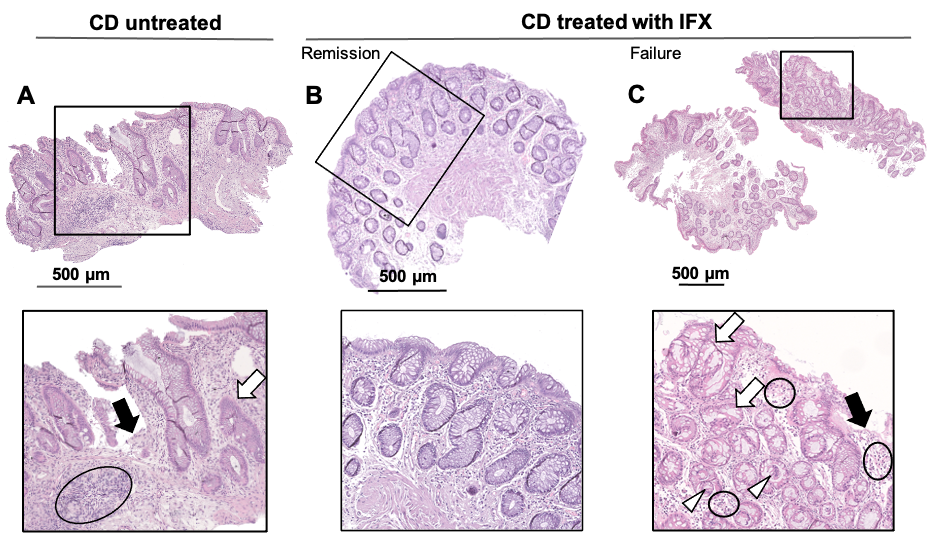
**

**Supplementary Figure 1. Histological scorings from CD patients. Related to Figure 1.** Histopathological features from biopsies of CD patients (A) untreated with IFX, (B) treated under remission and (C) treated failed. Ulcer (black arrows); Damaged crypts (white arrows); Crypts abscesses (white arrowheads), inflammatory infiltrates (black circles). Embedding: paraffin. Sections: 3 μm. Bars: 500 μm. Staining: Hematoxylin-Eosin.

**
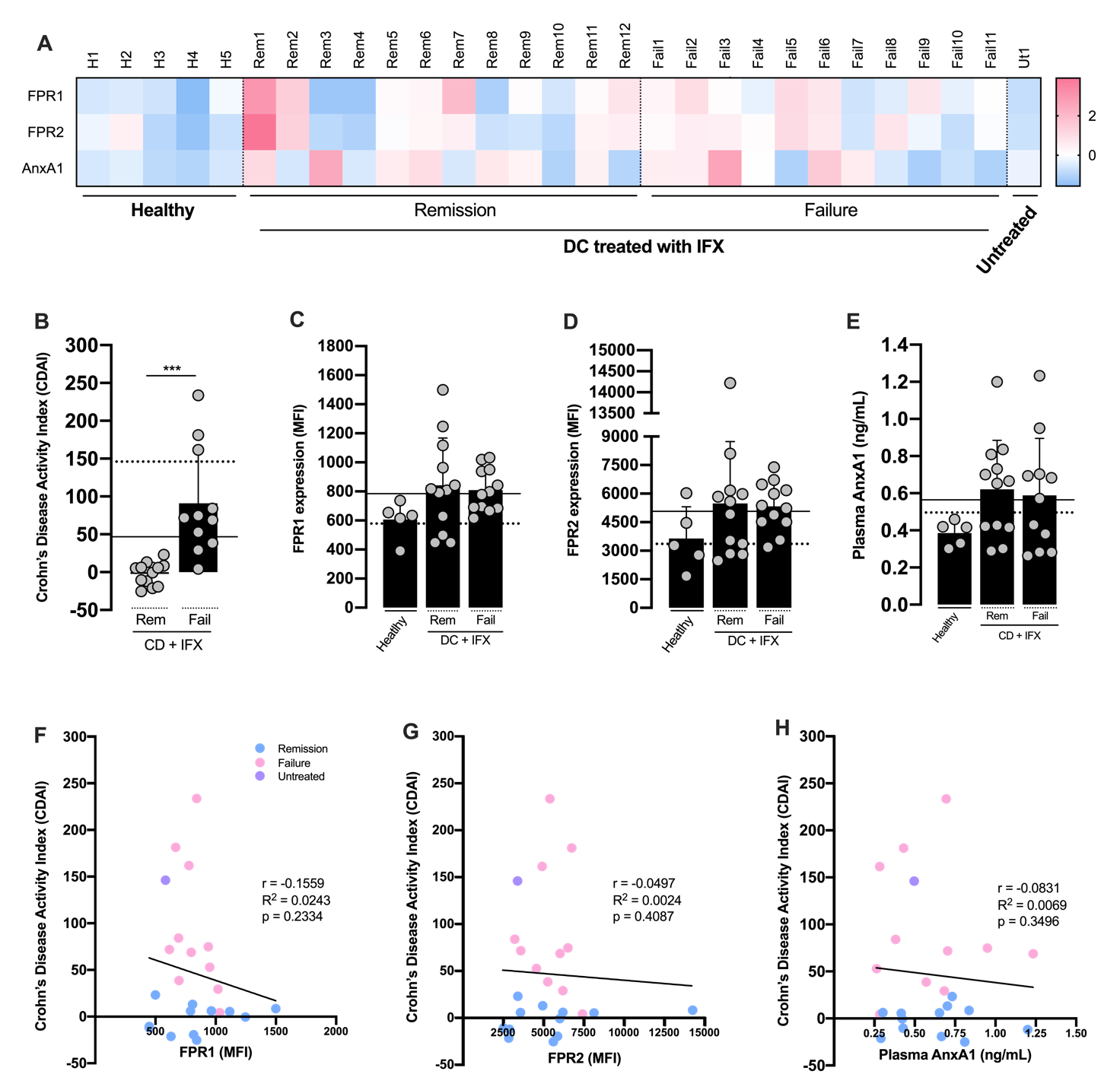
**

**Supplementary Figure 2. IFX does not change systemic levels of FPR1, FPR2 and AnxA1 in CD patients. Related to Figure 1.** (A) Heatmap based on the Z-scores of FPR1, FPR2 (circulating leukocytes) and AnxA1 (secreted in plasma). (B) Crohn’s Disease Activity Index (CDAI), based on Supplementary Table 2. Leukocytes MFI for (C) FPR1 and (D) FPR2. (E) Plasmatic AnxA1. Mean of all samples (continuous lines); Values from untreated patient (dashed lines). Correlation analysis between Crohn’s Disease Activity Index (CDAI) and circulating (F) FPR1, (G) FPR2 and (H) AnxA1. n=5 (Healthy donors), n=12 (Remission CD+IFX), n=11 (Failure CD+IFX), n=1 (Untreated CD patient). Results expressed as mean ± S.D.

**
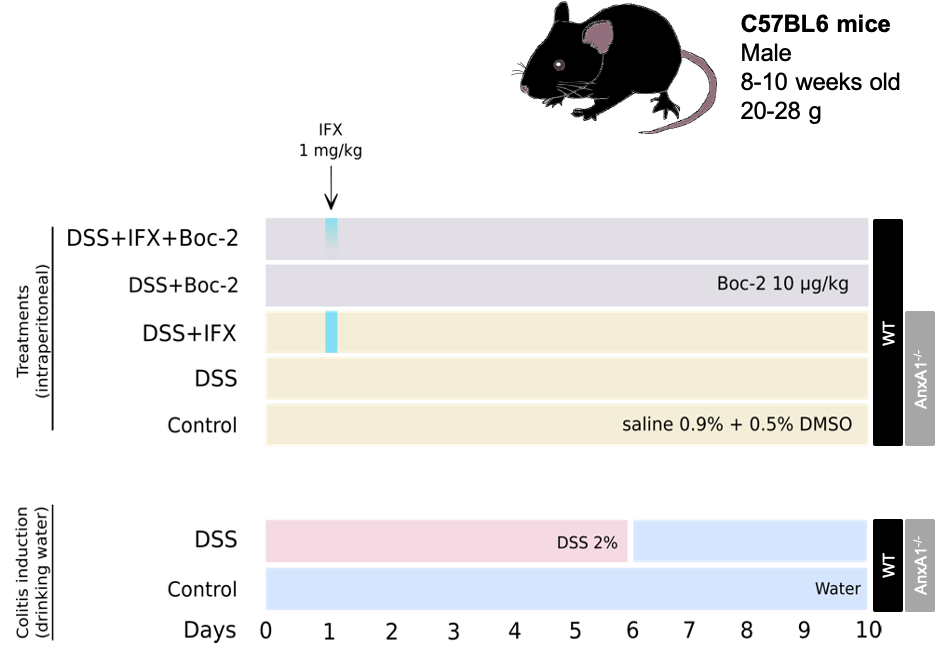
**

**Supplementary Figure 3. Design of *in vivo* experiments. Related to Figures 2-4.** Dextran sodium sulphate (DSS) colitis model was performed in wild-type (WT) and AnxA1-knockout (AnxA1^-/-^) C57BL6 male mice, 8-10 weeks old and weighting 20-28 g. DSS was administered to the drinking water at Day 0, changed for fresh solution at Days 2 and 4, and withdraw at Day 6. Mice were followed up until Day 10. Control and Boc-2 groups were treated daily with intraperitoneal injections of vehicle (saline 0.9% + 0.5% DMSO) or Boc-2 in vehicle (10 μg/kg). IFX groups received the treatment at Day 1.


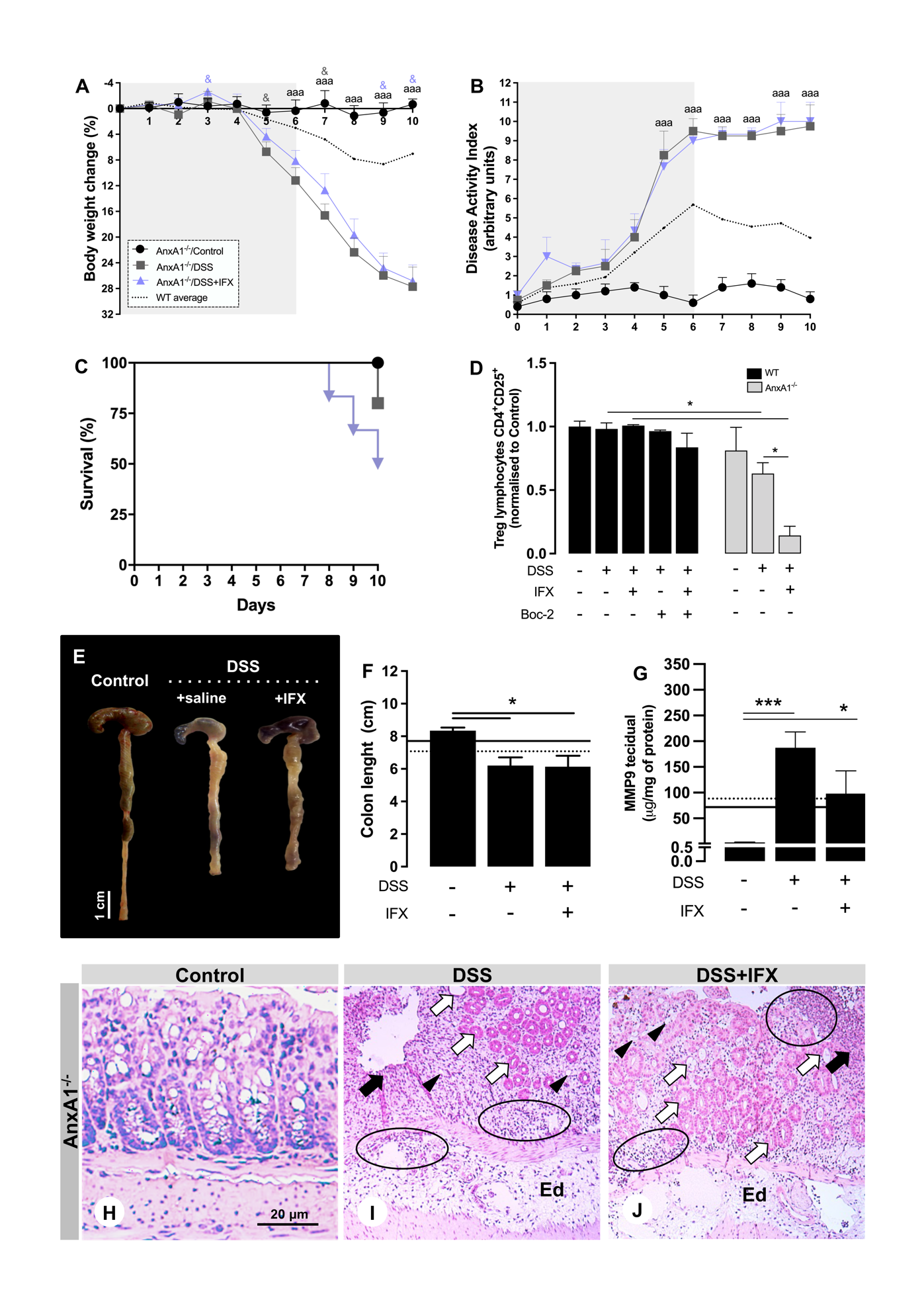


**Supplementary Figure 4. Absence of endogenous AnxA1 abrogates IFX resolutive effects on experimental colitis. Related to Figure 2.** (A) Loss of body weight. (B) Disease Activity Index (DAI). (C) Survival rates. (D) Relative percentages of Treg cells in the colon. (E and F) Large intestine length and anatomic changes. (G) MMP-9 levels in the colon. Mean of WT and AnxA1^-/-^ values (continuous lines); Mean of AnxA1^-/-^ mice (dashed lines). ^aaa^ p < 0.001 DSS vs Control; ^&^ p < 0.05 AnxA1^-/-^/DSS vs WT/DSS (grey) or AnxA1^-/-^/DSS+IFX vs WT/DSS+IFX (blue); * p < 0.05, *** p < 0.001. Results expressed as mean ± SEM. (H-J) Histopathology from AnxA1^-/-^ mice. Ulcer (black arrows); Altered crypts (white arrows); Vacuolar hydropic degeneration (black arrowheads); Oedema (Ed); inflammatory infiltrate (circles). Staining: Hematoxylin-Eosin. Embedding: paraffin. Sections: 3 μm. Bar: 20 μm. n = 3-5 mice/group.

**Supplementary Table 1. Related to Figure 1.**

| **Supplementary Table 1.** System for scoring microscopic parameters in patients with CD ^a^. | |
| --- | --- |
| **Microscopic grading** **^b^ for CD** **^a^** | |
| Epithelial damage | 0 = normal  1 = focal  2 = extensive |
| Histoarchitectural changes | 0 = normal  1 = moderate  2 = severe |
| Mononuclear cells in the lamina propria | 0 = normal  1 = moderate increase  2 = severe increase |
| Polymorphonuclear cells in the lamina propria | 0 = normal  1 = moderate increase  2 = severe increase |
| Neutrophils in the epithelium | 1 = surface epithelium  1 = cryptitis  2 = crypt abscess |
| Erosion or ulceration | 0 = no; 1 = yes |
| Granuloma | 0 = no; 1 = yes |
| ^a^ CD: Crohn’s disease;  ^b^ Microscopic grading: based in Laharie et al., 2011. | |

**Supplementary Table 2. Related to Figure 1.**

| **Supplementary Table 2.** Clinical parameters used to calculate the CD ^a^ Activity Index. | |
| --- | --- |
| **CDAI** **^b^** | |
| **Parameter** | **Score** |
| Average of liquid/soft stool occurrences during the last 7 days | (x2) |
| Average of abdominal pain intensity during the last 7 days | 0 = no pain  1 = mild pain  2 = moderate pain  3 = severe pain  (x5) |
| Average of wellbeing feeling during the past 7 days | 0 = good  1 = below the average  3= bad  4 = really bad  5 = terrible  (x7) |
| Anti-diarrheics in use | 0 = no  1 = yes  (x30) |
| Number of complications | - Arthritis or arthralgia - Iritis or uveitis - Erythema nodosum/pyoderma gangrenosum/aphthous ulcer - Anal fissures/fistula/perirectal abscesses - Fever during the last week (> 36.8°C)   (x20) |
| Abdominal mass | 0 = no  2 = questionable  5 = definite  (x10) |
| Haematocrit (Ht ^c^) | Male = 47- Ht  Female = 42 - Ht  (x6) |
| Percentage deviation from the usual body weight | 100 x ((1) - (current weight/ideal weight))  (x1) |
| ^a^ CD: Crohn’s disease;  ^b^ CDAI: CD Activity Index, adapted from Best et al., 1976;  ^c^ Ht: haematocrit. | |
